# Design and Manufacture of Millimeter-Scale 3D Transformers for RF-IC

**DOI:** 10.3390/mi13122162

**Published:** 2022-12-07

**Authors:** Haiwang Li, Kaiyun Zhu, Tiantong Xu, Kaibo Lei, Jingchao Xia

**Affiliations:** 1 National Key Laboratory of Science and Technology on Aero-Engine Aero-Thermodynamics, Beihang University, Beijing 100191, China; 2 Research Institute of Aero-Engine, Beihang University, Beijing 100076, China; 3 Beijing Microelectronic Technology Institute, Beijing 100076, China

**Keywords:** millimeter-scale 3D transformer, solenoidal, toroidal, MEMS, radio-frequency integrated circuits (RF-IC)

## Abstract

The development of radio-frequency integrated circuits (RF-IC) necessitates higher requirements for the size of microtransformers. This paper describes millimeter-scale 3D transformers in millimeter-scale, solenoidal, and toroidal transformers manufactured using Micro-electromechanical Systems (MEMS). Two through-silicon via (TSV) copper coils with a high aspect ratio are precisely interleaved on a reserved air core (magnet core cavity) with a vertical height of over 1 mm because of the thickness of the substrate, which increases the performance while reducing the footprint. The effects of the wire width, coil turns, magnetic core, and substrate on the performance of the two transformers are discussed through numerical simulations. When an air core is present, solenoidal transformers are better than toroidal transformers in terms of performance and footprint; however, the gap decreases when the size is reduced. Additionally, the magnetic core significantly improves the performance of the toroidal transformer compared to that of the solenoid. Thus, the toroidal transformer has a higher potential for further size reduction. The two types of transformers were then manufactured completely using MEMS and electroplating. This paper discusses the influence of various parameters on millimeter-scale 3D transformers and realizes processing in silicon, which provides the foundation for integrating transformers in a chip.

## 1. Introduction

The microtransformer plays a significant role in the development of electronic devices and is one of the main components of the information transfer module, which is an indispensable component of radio-frequency integrated circuits (RF-IC) [1,2,3]. The integrated components with transformers, such as power converters, voltage regulator converters, and isolators, perform the functions of transmitting power, transforming voltage, transmitting signal, and blocking interference [4,5,6,7,8]. The transformer occupies a large area, making it difficult to integrate it into an RF-IC. Thus, a high-performance miniature transformer that can be integrated with the RF-IC was designed to further reduce the size of the RF-IC and improve its integration [9].

Two types of microtransformers based on the coil structure have been reported. With planar helix coils [7,10,11,12], the primary and secondary coils form a spiral in a plane, which is difficult to integrate with a closed magnetic core and is too large to meet the needs of further miniaturization [9,11]. The other type includes 3D coils, usually divided into solenoidal and toroidal [9], and the central axes of which are generally coplanar. The general methods used to improve a 3D microtransformer include staggered winding, dense winding, and lamination core. Some of these methods have been reported in previous reports [13,14,15], such as a solenoid microtransformer using FeCoB multilayers as a sliced magnetic core and an integrated transformer with magnetic, thin films. However, the thickness of these transformers is still extremely small. If the dimensions in the thickness direction are exchanged with those in one direction on the plane, a higher inductance density can be obtained. Materials with high resistance are unsuitable for transformer coils [16]; hence, copper is a common coil material.

Generally, the inductance and quality factor, coupling coefficient, and maximum available gain are the main performance indexes of the transformer. An integrated core is the most direct way to improve the comprehensive performance of a transformer [9,10,12], although it produces some core loss. Moreover, proven methods to improve transformer performance include interleaving the primary and secondary coils, increasing the winding density, and using special core structures or materials. However, there are few studies on the influence of other structural parameters on transformer performance.

This study proposes two types of micro 3D transformers in-chip: solenoidal and toroidal. The influence of parameters such as the wire width, turns, and coil width on the performance and a comparison of the two types are also discussed. Subsequently, the Micro-electromechanical Systems (MEMS) manufacturing process of the transformer is described, and the reliability and stability of the two types of transformers are verified by X-ray microscopy and coils exposed after removing the silicon substrate.

## 2. Design and Modeling

**Figure 1:**
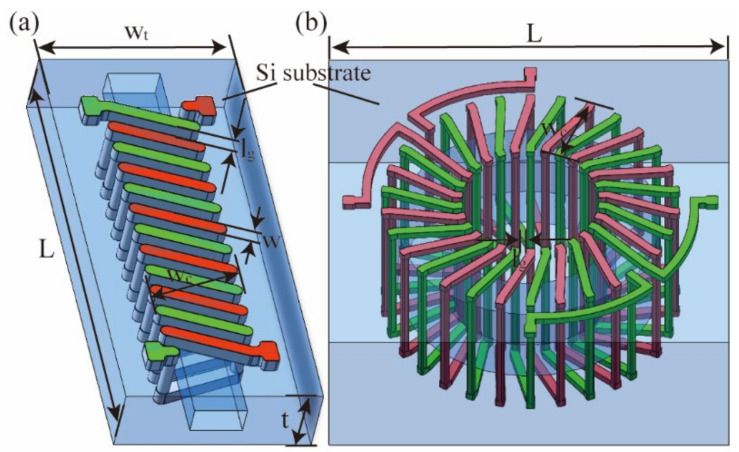
Models of the two transformer types. (**a**) Solenoidal and (**b**) toroidal.

Figure 1 shows the two structures—solenoidal and toroidal. The gap between the wires *l*_g_ is 100 μm, making the capacitance small enough to ensure a certain degree of isolation between wires while considering the machining error. The size of the transformer in the direction of the silicon substrate thickness *t* is 1000 μm, which is the thickness of the two wafers. Moreover, 1 μm thick silicon dioxide is placed between the coil and substrate to ensure insulation. The parameters selected for the wire width *w*, coil turns *n*, and coil width *w*_c_ are listed in Table 1. The edge of the substrate is 400 μm from the edge of the structure, with the areas of the two structures calculated using the modeling software *SolidWorks 2021* (Waltham, MA, USA).

Under the same parameters, the area of the toroidal transformer was larger than that of the solenoidal transformer.

## 3. Simulation and Analysis

**Figure 2:**
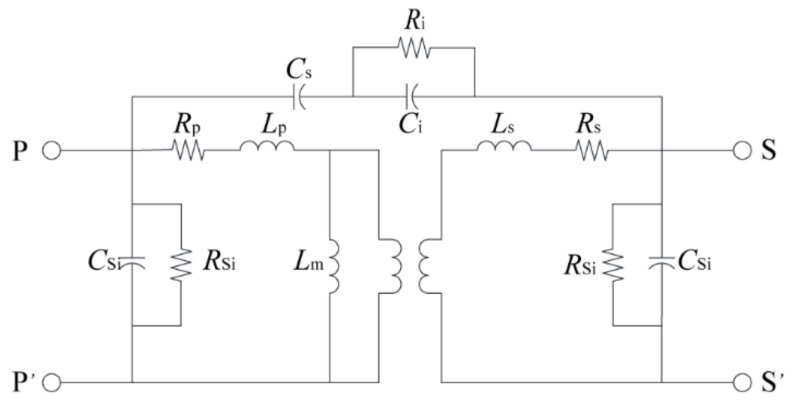
Equivalent circuit model of the 3D in-chip transformer.

A simple equivalent circuit model of the 3D in-chip transformer is shown in Figure 2. *R*_p_ and *L*_p_ are the resistance of the primary coil and leakage inductance generated, respectively, whereas *R*_s_ and *L*_s_ are the resistance and leakage inductance losses of the secondary coil, respectively. *L*_m_ is the excitation inductance, i.e., the equivalent inductance that produces magnetic flux. *C*_s_ is the equivalent capacitance between the adjacent wires of the primary and secondary coils, and *R*_i_ and *C*_i_ are the losses of their silicon substrates. *R*_Si_ and *C*_Si_ are the overall substrate losses, caused by the eddy current loss of the substrate and displacement current, among other factors. Through the two-port network constructed by this model, the parameters of the transformer in actual operation can be calculated.

The 1:1 model of all transformers in Table 1 was established in *HFSS. Solution Type* in *HFSS* was selected as *terminal*. One end of the two coils of the transformer was grounded, while excitation was added at the other ends [17]. As in the actual situation, the material of the substrate was set as Si with a conductivity of 5000 Ω/cm, and the coils and ground-signal-ground (GSG) were set as copper with an external layer of 1 um thick SiO_2_. In the simulation, the main performances of the transformer include inductance *L*, coupling coefficient *k*, and gain *G*, which can be calculated using the following formulas:

(1) $$L=\frac{Im\left(Z_{11\;\left(22\right)}\right)}{2\pi f}$$

(2) $$k=\sqrt{\frac{Im\left(Z_{12}\right)Im\left(Z_{21}\right)}{Im\left(Z_{11}\right)Im\left(Z_{22}\right)}}$$

(3) $$G=1+2\left(x-\sqrt{x^{2}+x}\right)$$

(4) x=Re(Z11)Re(Z22)−Re(Z12]2Re(Z12]2+Im(Z12]2

### 3.1. Simulation of Solenoidal Transformer with Air Core

**Figure 3:**
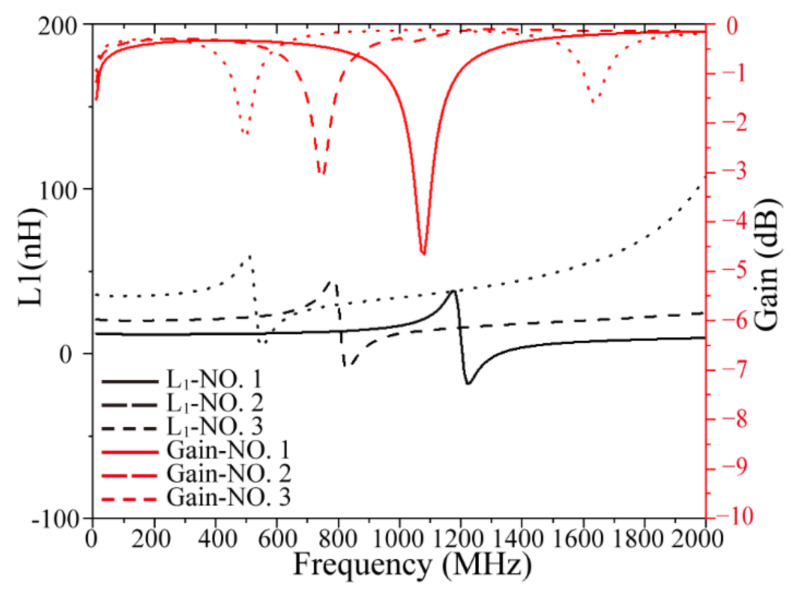
Calculated inductance and maximum available gain of solenoidal transformer with air core.

Table 2 lists the simulation results of the solenoidal transformer, including the inductance value, inductance density of the primary coil at 10 MHz, maximum coupling coefficient, and maximum available gain before the first resonance frequency of the transformer. The first resonance frequency of the gain was used to represent the working bandwidth of the transformer. In order to see the performance changes of transformers with different parameters more intuitively, calculated variation curves of the inductance and maximum available gain of some models are shown in Figure 3. Before the first resonant frequency, the inductance and gain of the three models are almost unchanged, and are about 12.39 nH, −0.32 dB; 20.87 nH, −0.29 dB; 35.97 nH, −0.29 dB, respectively. Then, when reaching the first resonance frequency, the inductance and gain of the transformer changes dramatically, so it is generally not suitable for use near this frequency. The first resonance frequencies of the three models are approximately 1.08 GHz, 0.74 GHz, and 0.5 GHz. Model No.3 has the highest inductance value 35.97 nH, inductance density 3.19 nH/mm^2^, and *k*_max_ 0.69 due to it having the greatest number of turns and the largest cross-sectional area; meanwhile, it had the smallest bandwidth. The increase in turns and coil width has the same effect on increasing the maximum coupling coefficient and maximum available gain in terms of coupling of the transformer with the air core, which improves the coupling coefficient by restraining more magnetic circuits and reducing the magnetic leakage. The increase in turns leads to a significant advance in the resonance frequency of the transformer and reduces the working bandwidth. Comparing models No.1 and No.5, we see that doubling the number of turns reduces the bandwidth by a factor of two.

An orthogonal experiment was used to analyze the influence of the structural parameters on the coupling coefficient of the transformer (Table 3)—the greater the extreme deviation, the greater the sensitivity. Notably, the wire width only has a certain influence on the inductance and inductance density, whereas the turns and coil width influence all aspects of the transformer’s performance. The inductance density is most sensitive to the wire width and decreases with an increase in the wire width.

### 3.2. Simulation of Toroidal Transformer with Air Core

**Figure 4:**
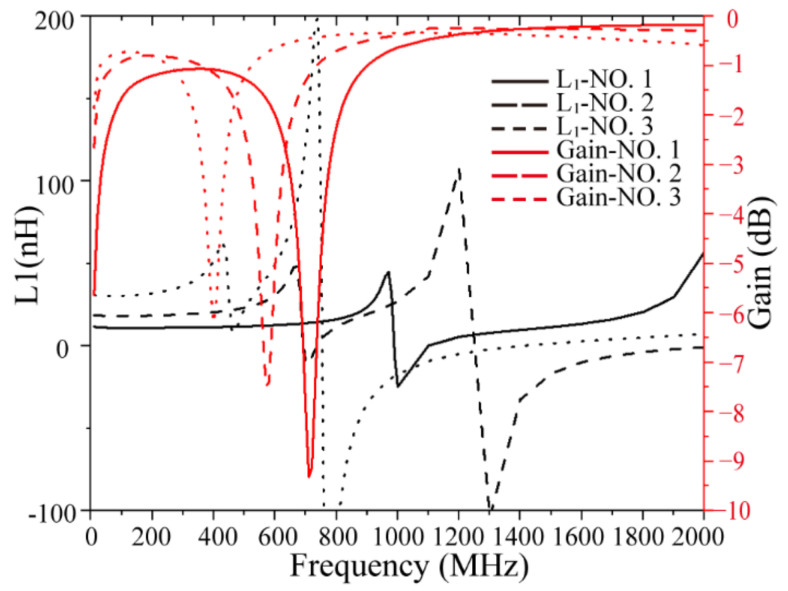
Inductance and maximum available gain of toroidal transformer with air core.

Table 4 and Figure 4 show the simulation results of the toroidal-structure transformer, and Table 5 presents the orthogonal experimental analysis. Almost all the performance parameters of the toroidal-structure transformers are lower than those of the solenoidal structures. The model with the maximum inductance and maximum inductance density is No. 3, but the values are 30.72 nH with 1.36 nH/mm^2^ for the toroidal structure and 35.97 nH with 3.19 nH/mm^2^ for the solenoidal structure, respectively. The maximum coupling coefficient and maximum available gain of No. 3 are 0.41 with −0.72 dB for the toroidal structure and 0.69 with −0.29 dB for the solenoidal structure.

However, when the overall size of the transformer is small, as in model No. 1, there is a reduced performance gap between the two structures. The larger coil spacing at the outer ring of the toroidal transformer coil is one of the main reasons for magnetic loss, with the spacing proportional to the overall size of the transformer. This loss can be reduced by reducing the size or adding a core. The influence of the wire width, turns, and coil width of the two structures on the transformer performance is similar; the difference is that the coupling parameters of the toroidal structure are more sensitive to turns, and the solenoidal structure is more sensitive to the coil width.

### 3.3. Effect of Substrate on the Transformer’s Performance

**Figure 5:**
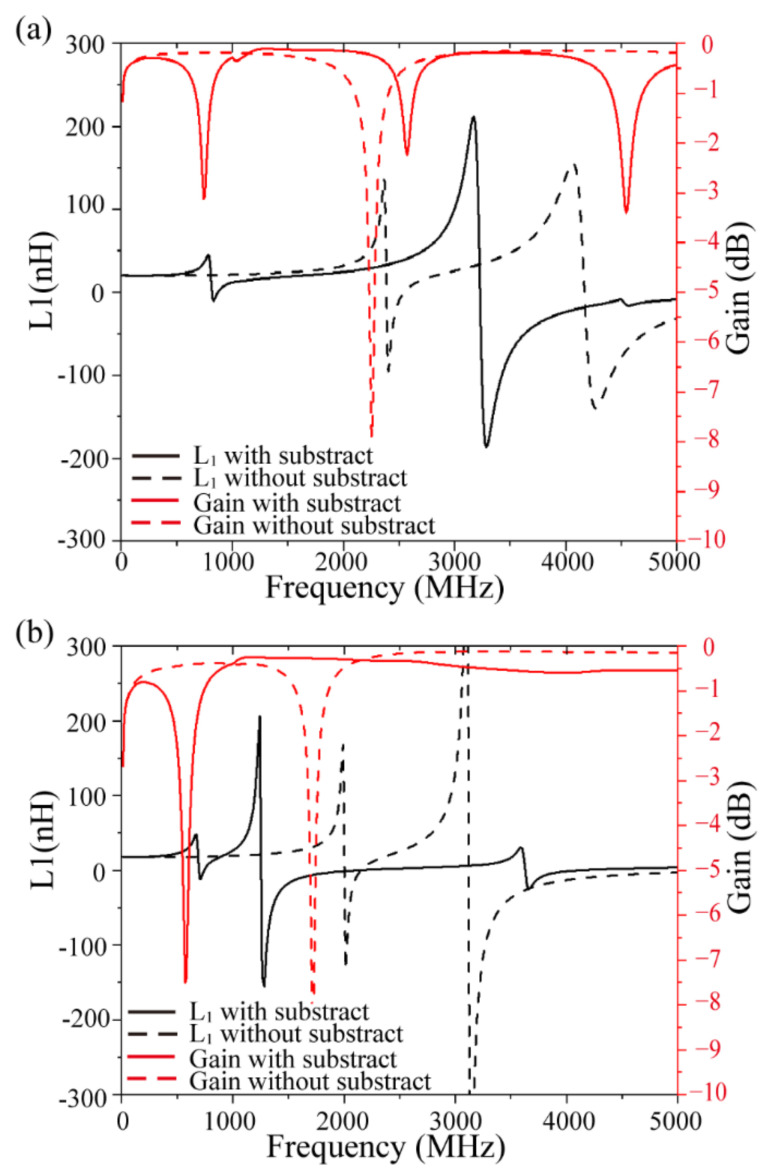
Effect of substrate on performance of transformer. (**a**) Solenoidal and (**b**) toroidal.

Figure 5 shows the effect of the substrate on the performance curve of the transformer. The resonant frequency of the transformer is greater than 2 GHz without a substrate, with the working bandwidth larger than that of the transformer with the substrate. After the substrate is added, the resonant frequency of the solenoidal transformer is reduced from 2.3 to 0.74 GHz, whereas that of the toroidal transformer is reduced from 3 to 0.57 GHz. It is inferred that the difference in the dielectric constant between silicon and air is a primary reason for the change in the resonance frequency. The parasitic capacitance increases and the resonance frequency decreases because the dielectric between the coils changes from air to silicon. However, within the working frequency range of the transformer, i.e., below 500 MHz, the substrate has little effect on the inductance and maximum available gain, which is explained by the fact that the magnetic core cavity (air core) in the substrate limits the large eddy current loss in the substrate when the frequency is below 500 MHz.

### 3.4. Simulation of Transformer with Ferrite Core

**Figure 6:**
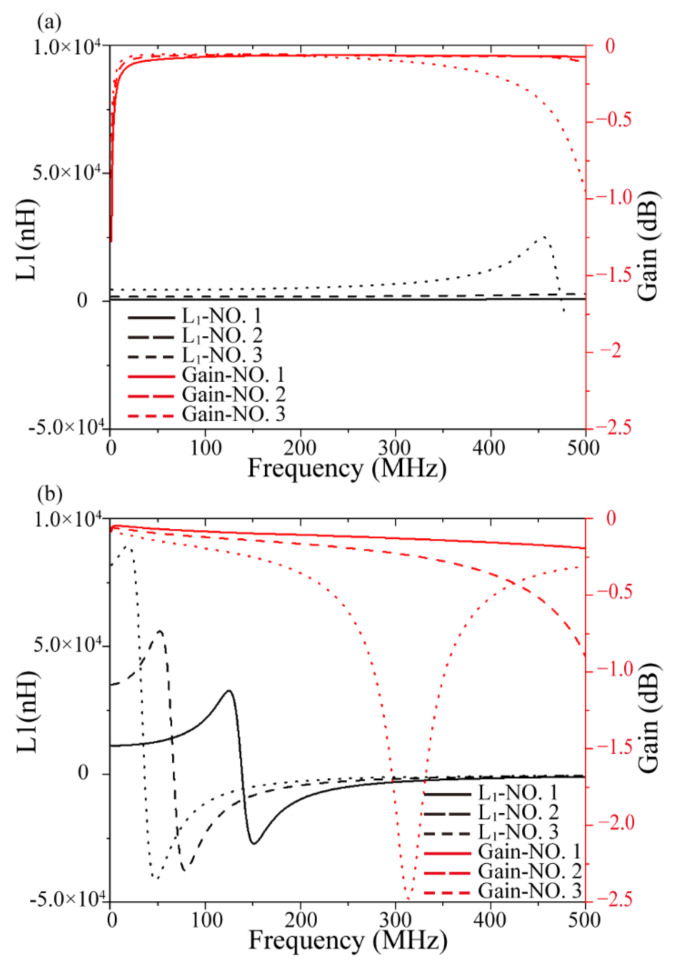
Inductance and maximum available gain of transformer with ferrite core. (**a**) Solenoidal and (**b**) toroidal.

Table 6 and Figure 6 show the simulation results for the solenoidal and toroidal transformers with ferrite as the core. Compared with the simulation results with the air core, the performance improvement of the two types of transformers is greater. In addition, the inductance, coupling coefficient, and maximum available gain are increased, whereas the first-order resonance frequency moved forward, implying that the working bandwidth is narrowed. For the solenoidal transformer with a wire width of 80 μm, turn number of 10, and coil width of 1000 μm, the primary-side inductance of the transformer changed from 35.97 to 474.22 nH after adding the magnetic core, a 13-fold increase. For the toroidal transformer, the inductance changed from 30.72 nH to 8.19 μH, a 267-fold increase. Simultaneously, the wire width, turns, and coil width have little influence on the coupling performance when the magnetic core is added. However, an increase in the number of turns and coil width can still increase the inductance.

## 4. Fabrication

**Figure 7:**
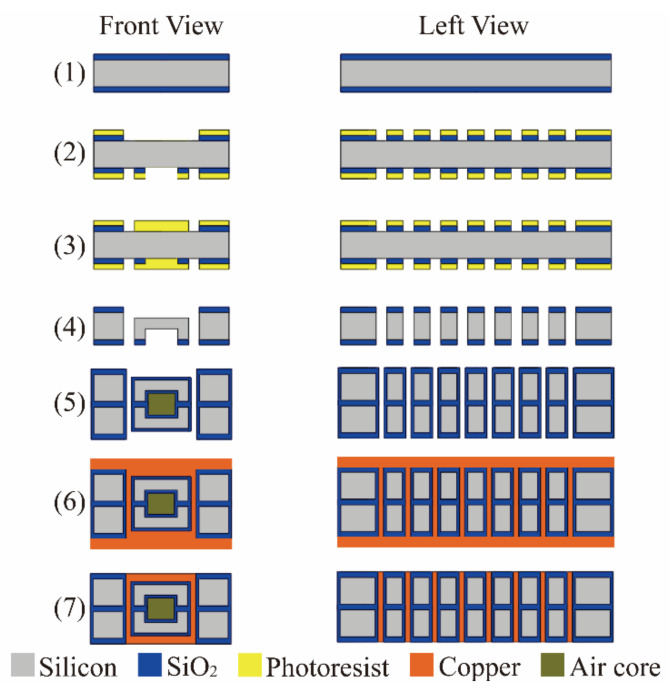
Brief processing flow of in-chip transformer. (1)–(7) respectively correspond to the 7 steps in the text.

The fabrication method of the transformer was reported in our previous work [18,19,20]. The fabrication process comprised seven stages, as shown in Figure 7:

(1) The substrate consisted of two 500 μm silicon wafers with 2 μm oxidations on both sides;
(2) S1813 photoresist was coated on both sides of the silicon wafer. The photoresist covering the horizontal trench (on both sides of the wafer) and air core (on bottom of the wafer) was exposed to ultraviolet light (10 mJ/cm^2^, 3 s), which would be removed in NMD developer (about 2 min). Then, the wafer was soaked in buffered oxide etchant (BOE) for about 20 min to remove the SiO_2_ covering the two surfaces of vertical vias;
(3) Cleaned the wafer with Piranha solution (98% H_2_SO_4_:30% H_2_O_2_ = 3:1, about 8 min) to remove the other photoresist. AZ4620 photoresist was coated on both sides of the wafer. The photoresist covering the two surfaces of through-hole was exposed to ultraviolet light (10 mJ/cm^2^, 13 s), which would be removed in AZ4620 developer (AZ4620:H_2_O = 1:3, about 2.5 min);
(4) Deep reaction ion etching (DRIE) was used to etch the through-hole and air core from the bottom of wafer. Stopped etching when the air core reached the set depth. Etched the through-hole from the top of wafer until the through-hole formed completely. Cleaned the wafer with Piranha solution. Etched the horizontal trench from the top of the wafer to reach the set depth;
(5) Soaked the wafers in HF solution (40% HF:H_2_O = 1:10) for about an hour to remove SiO_2_. Cleaned the wafer with Piranha solution. Si–Si direct bonding was used to combine the air core side of two wafers to form the entire coil slot and air core. Then, they were thermally oxidized to form an SiO_2_ insulating layer;
(6) Cleaned the wafer with Piranha. Sputtering copper-seed layer (about 800 nm) on one side of bonded wafer by magnetron sputtering. Filled the entire bottom horizontal trench and through-hole with copper from bottom to top by electroplating. Cleaned the wafer with acetone. Sputtering copper-seed layer (about 800 nm) on the other side of wafer magnetron sputtering. Filled the horizontal trench coil with copper by electroplating. Then, the whole electroplating process ended;
(7) Excess copper was removed by lapping and polishing to complete the process.

**Figure 8:**
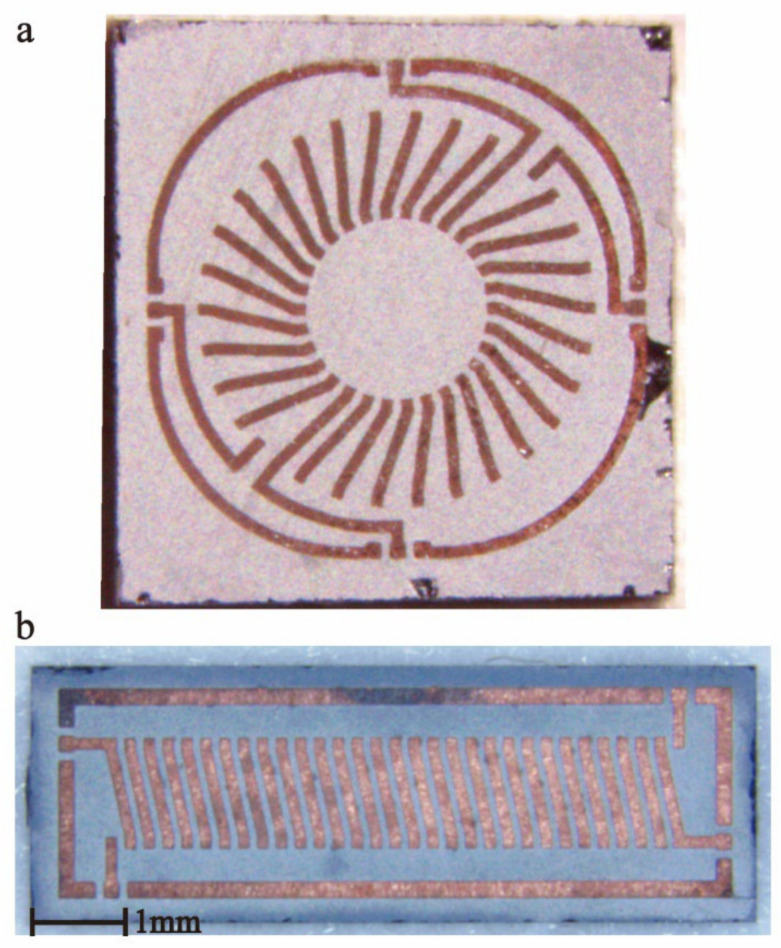
(**a**) Toroidal and (**b**) solenoidal structure transformers.

As shown in Figure 8, the toroidal and solenoidal transformers were obtained through the above process. The greater the number of turns, the greater the possibility of defects; thus, 15-15 turns with a relatively large number of turns were chosen to demonstrate our actual processing capacity. Finally, the toroidal and solenoidal transformers were 5.2 × 5.2 × 1 mm and 2.5 × 7 × 1 mm, respectively.

**Figure 9:**
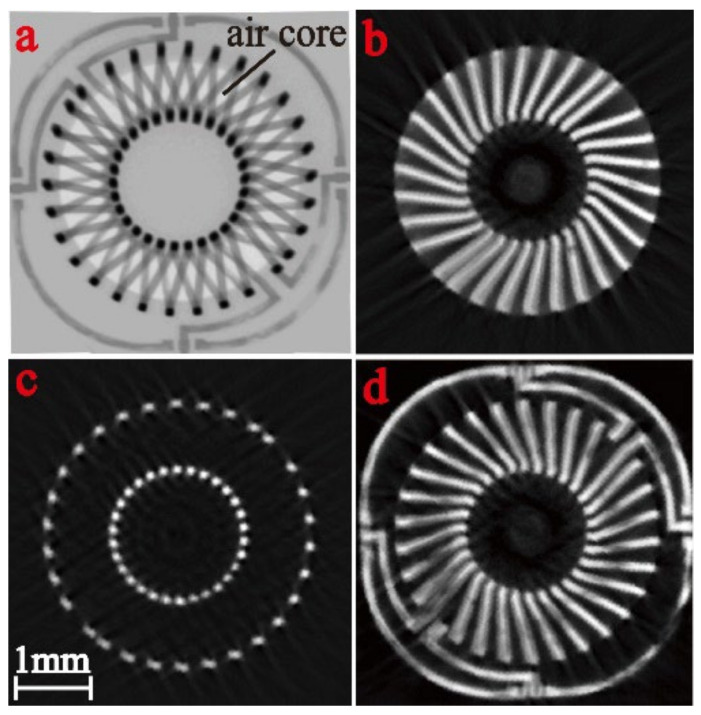
X-ray microscope view of a toroidal transformer. (**a**) Superposition of field of views in thickness direction (*t* direction). Views of the cross-section (**b**) 50 µm; (**c**) 500 µm; and (**d**) 950 µm from the bottom.

**Figure 10:**
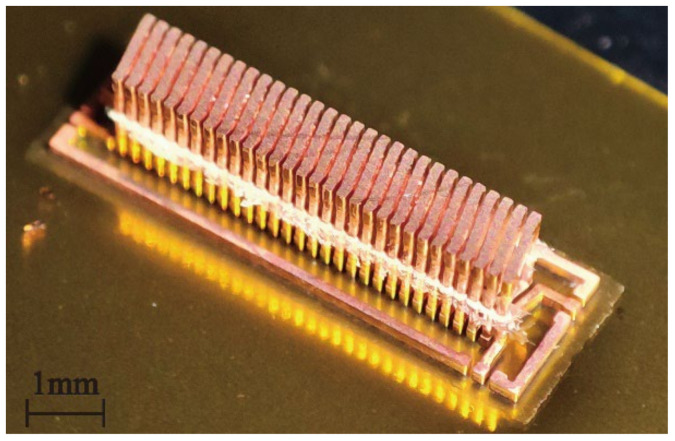
Exposed coils after removal of silicon substrate.

We used two methods to verify the reliability of our process. (1) First, an X-ray microscope was used to observe the overall structure of transformers in silicon (Figure 9). Through sections at different heights, we could see that all the copper coils, air core, and silicon had no defects, which proved that our fabrication process was reliable. (2) Simultaneously, we removed the silicon substrate by DRIE in Figure 10 and exposed the entire electroplated copper coils for direct observation. Owing to the high density of the electroplating, the structure remained stable under a slight stir.

The four coils of the two transformers had a DC resistance of approximately 0.15 Ω.

**Figure 11:**
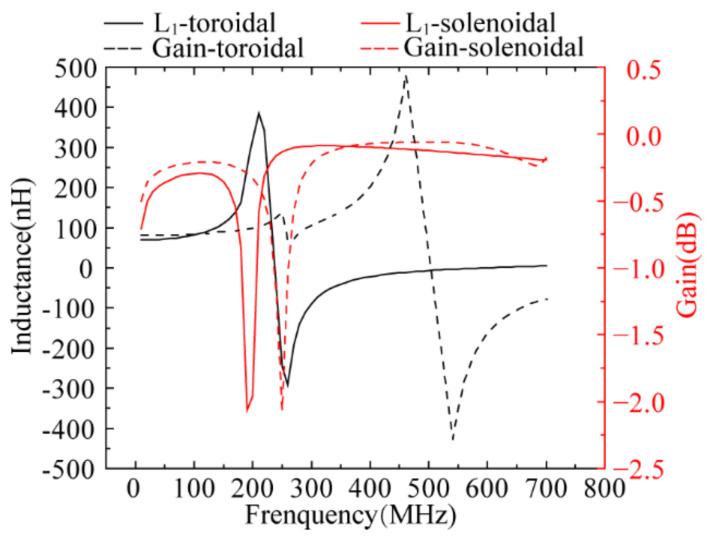
Simulation results of the two transformers.

Then, the performances of the two transformers were simulated. Table 7 and Figure 11 shows the detailed results. Under the same *w*, *n*, and *w*_c_, the solenoidal transformer has a better performance than the toroidal transformer.

## 5. Conclusions

In this study, we presented an in-chip solenoid microtransformer with coils embedded in a silicon substrate, which uses the size of the silicon wafer in the thickness direction to ensure it has a large cross-sectional area and does not occupy too much substrate surface area.

For air core transformers, increasing turns and coil width will increase inductance while reducing bandwidth; thus, decreasing the wire width will increase the inductance density. The solenoidal transformer was better than the toroidal transformer in terms of performance and footprint, but the gap decreased when the size was reduced. The maximum inductance was 35.97 nH, the maximum inductance density was 3.19 nH/mm^2^, and the maximum coupling coefficient was 0.69 in the simulation results.

A magnetic core significantly improves the performance of a toroidal transformer compared to that of a solenoidal transformer, which can be explained by the fact that the core is completely wrapped by coils. The inductance was 13-fold better for the solenoidal structure and 267-fold better for the toroidal structure. Thus, the toroidal transformer has a higher potential for further size reduction.

Due to the resistance and parasitic capacitance of the substrate, the existence of the substrate greatly reduces the bandwidth. Thus, it is an important means to improve the performance of microtransformers to use silicon materials with higher resistance as substrate or without substrate.

Subsequently, 15-15 turns of the solenoidal and toroidal transformers were manufactured by MEMS, which is compatible with CMOS. Finally, the observed internal structures of the transformer, which were obtained using an X-ray microscope and the coils after removing the silicon substrate, prove that the fabrication processing is reliable and that the transformer has high structural strength.

In future studies, we will manufacture transformers with various parameter settings to represent more testing details of our transformers.

## Figures and Tables

**Table 1:** Structure parameters of solenoidal and toroidal transformers.

No.	*w*/μm	*n*	*w*_c_/μm	Area (Toroidal) /mm^2^	Area (Toroidal) /mm^2^
1	80	5	500	5.79	10.13
2	80	7	750	7.91	15.27
3	80	10	1000	11.28	22.56
4	100	7	1000	9.60	20.23
5	100	10	500	10.01	15.04
6	100	5	750	7.00	14.05
7	120	10	750	12.00	20.30
8	120	5	1000	8.38	18.59
9	120	7	500	8.51	12.87

**Table 2:** Simulation result of solenoidal transformer with air core.

No.	*L*/nH	*L*_desity_/(nH/mm^2^)	*k*_max_	*G*_max_/dB	Bandwidth/GHz
1	12.39	2.14	0.54	−0.32	1.08
2	20.87	2.64	0.63	−0.29	0.74
3	35.97	3.19	0.69	−0.29	0.5
4	23.49	2.45	0.66	−0.26	0.66
5	21.53	2.15	0.61	−0.34	0.63
6	14.42	2.06	0.58	−0.27	0.95
7	24.02	2.00	0.63	−0.32	0.55
8	16.12	1.92	0.61	−0.25	0.84
9	15.09	1.77	0.57	−0.31	0.82

**Table 3:** Extreme deviation analysis of solenoidal transformer with air core.

Extreme Deviation	Wire Width	Turns	Coil Width
R(*L*)	14.00	38.58	26.57
R(*L*_desity_)	2.27	1.22	1.50
R(*k*_max_)	0.05	0.21	0.25
R(*G*_max_)	0.03	0.11	0.18
R(*Bandwidth*)	0.11	1.19	0.53

**Table 4:** Simulation result of toroidal transformer with air core.

No.	*L*/nH	*L*_desity_/(nH/mm^2^)	*k*_max_	*G*_max_/dB	Bandwidth/GHz
1	11.67	1.15	0.20	−1.06	0.71
2	18.63	1.22	0.31	−0.80	0.57
3	30.72	1.36	0.41	−0.72	0.4
4	20.61	1.02	0.31	−0.79	0.51
5	19.04	1.27	0.33	−0.90	0.46
6	13.34	0.95	0.21	−1.02	0.64
7	22.81	1.12	0.37	−0.76	0.41
8	14.83	0.80	0.27	−0.42	0.65
9	13.30	1.03	0.24	−1.02	0.55

**Table 5:** Extreme deviation analysis of toroidal transformer with air core.

Extreme Deviation	Wire Width	Turns	Coil Width
R(*L*)	10.08	32.73	22.15
R(*L*_desity_)	0.78	0.85	0.27
R(*k*_max_)	0.12	0.33	0.32
R(*G*_max_)	0.07	0.43	0.22
R(*Bandwidth*)	0.07	0.73	0.16

**Table 6:** Simulation result of ferrite core transformer.

No.	Solenoidal Structure			Toroidal Structure		
	*L*/nH	*k*_max_	*G*_max_/dB	*L*/uH	*k*_max_	*G*_max_/dB
1	82.76	0.92	−0.062	1.13	0.99	−0.045
2	194.22	0.95	−0.058	3.51	1.00	−0.054
3	474.22	0.97	−0.056	8.19	1.00	−0.065
4	225.29	0.96	−0.056	4.58	1.00	−0.050
5	349.67	0.97	−0.049	2.72	1.00	−0.062
6	100.36	0.93	−0.060	2.01	0.99	−0.041
7	267.12	0.96	−0.049	5.14	1.00	−0.054
8	115.95	0.94	−0.060	2.60	0.46	−0.651
9	168.77	0.95	−0.052	1.58	0.99	−0.048

**Table 7:** Simulation results of the two transformers.

	*L*/nH	*k*_max_	*G*_max_/dB	Bandwidth/MHz
Toroidal	About 72	0.69	−0.088	190
Solenoidal	About 90	0.81	−0.060	250

## Data Availability

Not applicable.
